# Neuronal and Astrocytic Morphological Alterations Driven by Prolonged Exposure with Δ9-Tetrahydrocannabinol but Not Cannabidiol

**DOI:** 10.3390/toxics10020048

**Published:** 2022-01-21

**Authors:** Elisa Landucci, Costanza Mazzantini, Daniele Lana, Maria Grazia Giovannini, Domenico E. Pellegrini-Giampietro

**Affiliations:** Department of Health Sciences, Section of Clinical Pharmacology and Oncology, University of Florence, Viale Pieraccini 6, 50139 Florence, Italy; costanza.mazzantini@unifi.it (C.M.); daniele.lana@unifi.it (D.L.); mariagrazia.giovannini@unifi.it (M.G.G.); domenico.pellegrini@unifi.it (D.E.P.-G.)

**Keywords:** THC, CBD, PSD95, organotypic hippocampal slice, neuron, astrocyte, toxicity, clasmatodendrosis

## Abstract

Cannabis derivatives are largely used in the general population for recreational and medical purposes, with the highest prevalence among adolescents, but chronic use and abuse has raised medical concerns. We investigated the prolonged effects of Δ9-tetrahydrocannabinol (THC) and cannabidiol (CBD) in organotypic hippocampal slices from P7 rats cultured for 2 weeks. Cell death in the CA1 subregion of slices was quantified by propidium iodide (PI) fluorescence, pre-synaptic and post-synaptic marker proteins were analysed by Western blotting and neurodegeneration and astrocytic alterations by NeuN and GFAP by immunofluorescence and confocal laser microscopy. The statistical significance of differences was analysed using ANOVA with a post hoc Dunnett w-test (PI fluorescence intensities and Western blots) or Newman–Keuls (immunohistochemistry data) for multiple comparisons. A probability value (*P*) of < 0.05 was considered significant. Prolonged (72 h) THC or CBD incubation did not induce cell death but caused modifications in the expression of synaptic proteins and morphological alterations in neurons and astrocytes. In particular, the expression of PSD95 was reduced following incubation for 72 h with THC and was increased following incubation with CBD. THC for 72 h caused disorganisation of CA1 stratum pyramidalis (SP) and complex morphological modifications in a significant number of pyramidal neurons and in astrocytes. Our results suggest that THC or CBD prolonged exposure induce different effects in the hippocampus. In particular, 72 h of THC exposure induced neuronal and glia alterations that must draw our attention to the effects that relatively prolonged use might cause, especially in adolescents.

## 1. Introduction

Cannabis has been widely used for thousands of years, both for recreational and medical purposes. Currently, cannabis derivatives are still largely used in the general population, with the highest prevalence among adolescents [1], but chronic use and abuse have raised medical concerns [2]. Cannabis can induce craving [3], dependence [4], and drug-seeking behaviour [5]. Recent developments in molecular and cellular neurobiology have provided new tools to understand in detail the mechanisms by which cannabinoids and especially ∆9-tetrahydrocannabinol (THC) produce long-lasting functional changes in the brain [6].

In humans and animal experimental models, it has been shown that chronic exposure to THC during adolescence produces long-term behavioural alterations that share similarities with certain symptoms of psychiatric and neurodevelopmental disorders [7,8]. However, little is known about the structural, functional, and molecular mechanisms underlying these deficits [9]. CBD mitigates the long-term behavioural alterations induced by THC chronic exposure in adolescent female rats as well as long-term changes in CB1 receptors and microglia activation in the prefrontal cortex (PFC) [10]. In addition, repeated administration of a CBD/THC combination, reminiscent of “light cannabis” (CBD: THC in a 33:1 ratio; total THC 0.3%), induces long-term adverse effects on cognition and leads to anhedonia [10]. Repeated exposure to THC in mice produces a decrease in the number of visits to Intelli Cage corners, a marker of reduced locomotor activity [11,12,13]. Clinical and experimental studies have shown that repetitive THC treatment induces behavioural tolerance, which coincides with rapid downregulation and desensitisation of cannabinoid receptor binding sites in several brain areas of the mesocorticolimbic circuitry and cerebellum [14,15]. Chronic THC affects schedule-induced drinking development, confirming that it can disrupt learning, possibly causing alterations in time estimation. In addition, chronic THC leads to sensitisation of animals when they are re-exposed to the drug after long periods without drug exposure [16]. These behavioural effects following prolonged exposure to THC and CBD, however, are not corroborated by functional electrophysiological synaptic plasticity experiments performed following prolonged incubation to cannabinoids.

The production of new synapses or reorganisation of existing synapses, including modifications in proteins that are dynamically regulated at pre- and post-synaptic sites are necessary for complex brain functions [17,18]. THC exposure during adolescence induces changes in glutamate synapse and glial cells [1]. In rats treated with THC for 10 days, Rubino and colleagues [19] observed impaired spatial memory and decreased expression of the post-synaptic marker PSD95 and of astrocytic GFAP in the hippocampus. Chronic exposure to CP55,940 (a synthetic cannabinoid agonist) alters both the morphology of pyramidal neurons and the expression of PSD95 protein in the prefrontal cortex (PFC) and induces plasticity changes in the hippocampus–PFC network of adult rats [9]. The modifications driven by THC on the hippocampus result in secondary effects on learning and attenuate the increased transcription of neuroplasticity markers observed during the training of control animals [20]. Suarez and colleagues [21] showed that pre- and perinatal THC exposure causes long-lasting changes in GFAP expression. The aforementioned treatment precisely interferes with astrocytic maturation by disrupting normal cytoskeletal formation, as indicated by the lower GFAP expression and its irregular arrangement in the cytoplasm observed at all ages studied [22].

In this study, we treated organotypic hippocampal slices for 24 or 72 h with either THC or CBD to understand the possible toxic effects of prolonged cannabinoids exposure and evaluate possible alterations in neuroplasticity. For this reason, we analysed the levels of both pre-synaptic and post-synaptic marker proteins (synaptophysin and PSD95, respectively) by Western blotting, and we investigated neurodegeneration and astrocytic alterations by immunohistochemistry for NeuN and GFAP, respectively.

## 2. Materials and Methods

### 2.1. Animals

Wistar rat pups of 7 days of age of both sexes were used (Charles River, MI, Italy). Rats, housed at 23 ± 1 °C under a 12 h light–dark cycle with lights on at 07:00, were fed a standard laboratory diet with ad libitum access to water. The experimental protocols were approved by the Animal Care Committee of the Department of Health Sciences, University of Florence (17E9C.N.GSO/2021).

The ethical policy of the University of Florence on the use of laboratory animals is in accordance with the Directive 2010/63/EU and with the Italian DL 26/2014 “protection of animals used for scientific purposes”. Accordingly, we fulfilled the principle of 3Rs. The experimental procedures were conducted in accordance with the ARRIVE guidelines and were authorised by the Italian Ministry of Health.

### 2.2. Materials

Cannabidiol (CBD) was purchased from Tocris Cookson (Bristol, UK). The medium for tissue cultures was purchased from Gibco-BRL (San Giuliano Milanese, MI, Italy), insert and Δ9-tetrahydrocannabinol (THC) were purchased from Sigma (St. Louis, MO, USA).

### 2.3. Preparation of Rat Organotypic Hippocampal Slice Cultures

**Figure 1:**
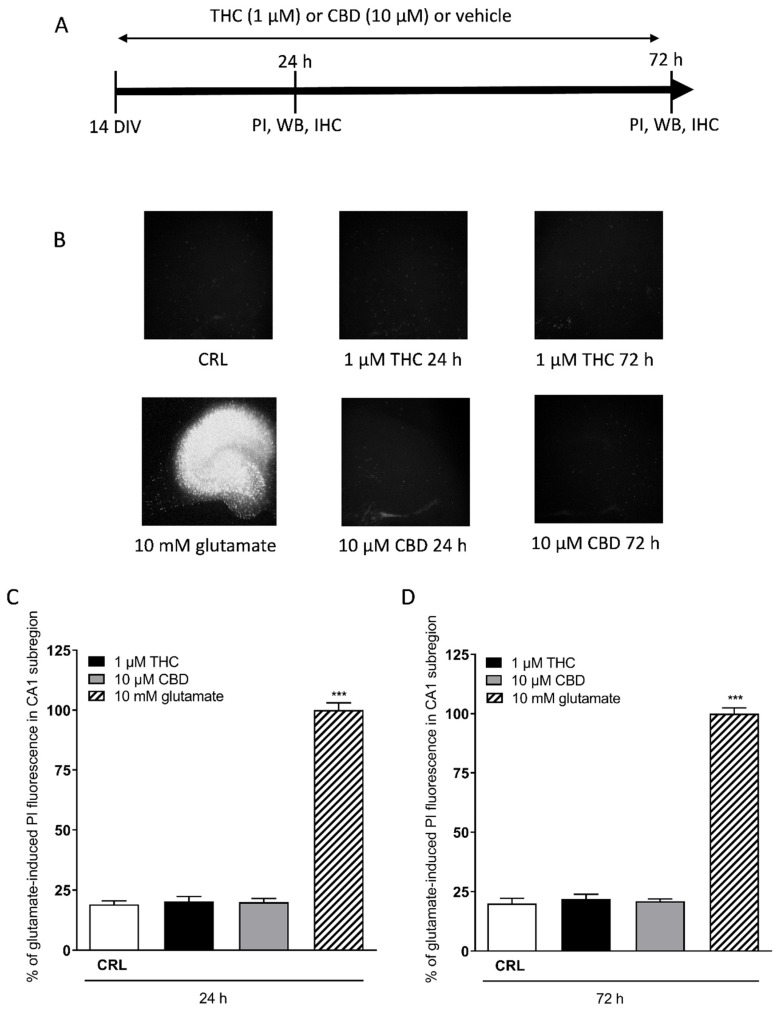
Effects of THC or CBD incubation in organotypic hippocampal slices. (**A**) Experimental protocols. (**B**) Top left: hippocampal slices under normal conditions (background PI fluorescence). Bottom left: slice exposed to 10 mM glutamate, displaying intense PI labelling in all sub-regions. Hippocampal slices incubated with 1 µM THC 24 h (top centre), 10 µM CBD 24 h (bottom centre). Slices incubated with 1 µM THC 72 h (top right), 10 µM CBD 72 h (bottom right). (**C**,**D**) Quantitative analysis at 24 h (**C**) and 72 h (**D**) incubation with cannabinoids. Bars represent the mean ± SEM of at least four experiments. *** *p* < 0.001 vs. control (CRL) (ANOVA + Dunnett’s test).

Organotypic hippocampal slice cultures were prepared as previously reported [23]. Briefly, hippocampi of Wistar rat pups were removed from the brains, and transverse slices (420 µm) were prepared by a McIlwain tissue chopper. After microscope selection, the slices were transferred onto inserts (Millicell-CM PICM03050; Millipore, Milan, Italy; four slices per insert), which were placed in six-well tissue culture plates containing 1.2 mL medium per well. The normal medium consists of 50% Eagle’s minimal essential medium, 25% heat-inactivated horse serum, 25% Hanks’ balanced salt solution, 5 mg/mL glucose, 2 mM L-glutamine, and 3.75 mg/mL amphotericin B. Slices were maintained at 37 °C in an incubator in atmosphere of humidified air and 5% CO_2_ for 2 weeks. The slices were exposed for 24 h or 72 h to 1 µM of THC and 10 µM of CBD; the medium was changed every day (Figure 1A).

The slices were incubated with propidium iodide (5 μg/mL) for cell death evaluation by using an inverted fluorescence microscope. Images were analysed using morphometric analysis software (ImageJ; NIH, Bethesda, MD, USA) by measuring the optical density of PI fluorescence (the glutamate fluorescence was reported as 100%). THC was dissolved in methanol, CBD was dissolved in dimethyl sulfoxide (DMSO), and both were stored at −20 °C. For the experiments, they were diluted in cell culture medium. The maximal final solvent concentration for the drugs was 0.1% (*v*/*v*) DMSO or 0.3% (*v*/*v*) methanol. Slices exposed to equimolar concentrations of DMSO or methanol alone did not show any significant effects (data not shown).

### 2.4. Western Blot Analysis

Western blotting was conducted as previously reported [24]. Each sample consists of four slices that were dissolved in 1% SDS. The total protein levels were quantified by BCA (bicinchoninic acid) protein assay. Lysates (20 μg/lane of protein) were resolved by electrophoresis on a 4–20% SDS-polyacrylamide gel (Bio-Rad Laboratories, Hercules, CA, USA) and transferred onto nitrocellulose membranes. After blocking 1 h with TBS-T containing 5% non-fat dry milk, the blots were incubated overnight at 4 °C with monoclonal-mouse antibody against PSD95 (from Cell Signaling Technology, Beverly, MA, USA) and monoclonal-mouse antibody against Synaptophysin (Sigma-Merk, Darmstadt, Germany), both diluted 1:1000 in TBS-T containing 5% bovine serum albumin. β-actin was used as a loading control (monoclonal antibody purchased from Sigma, St Louis, MO, USA). Immunodetection was performed with HRP-conjugated secondary antibodies (1:2000 anti-mouse, anti-rabbit, or anti-goat IgG from donkey, Amersham Biosciences, Amersham, UK) in TBS-T containing 5% non-fat dry milk. After extensive washings, the reactive bands were detected using chemiluminescence (ECLplus; Euroclone, Padova, Italy). The quantitative analysis was obtained with Quantity One analysis software (Bio-Rad, Hercules, CA, USA). Results are presented as the mean ± standard error of the mean (SEM) of different gels and expressed as AU, which depicts the ratio between levels of target protein expression and β-actin normalised to basal levels.

### 2.5. Fluorescence Immunohistochemistry and Quantitative Analysis

After the 24 h or 72 h incubation with either THC or CBD, the organotypic slices were fixed overnight in cold paraformaldehyde dissolved in PBS buffer (4%). The following day, slices were incubated for at least 48 h in cold solution of sucrose in PBS (18%). Immunofluorescence staining was performed with the free-floating protocol according to Landucci et al. and Gerace et al. [25,26].

First day: slices were blocked with blocking buffer (BB containing 10% normal goat serum) for 1 h, and were incubated O/N at 4 °C with a mouse anti-NeuN to immunostain neurons dissolved in BB (1:400; product code #MAB377, Millipore, Billerica, MA, USA).

Second day: slices were incubated for 2 h at the RT in the dark with AlexaFluor 555 donkey anti-mouse IgG (1:400 in BB; product code #A31570, Thermo Fisher Scientific, Waltham, MA, USA). Astrocytes were visualised using a mouse anti-GFAP antibody conjugated with the fluorochrome AlexaFluor 488 for 2 h at room temperature in the dark (1:500 in BB; product code #MAB3402X, Millipore). The slices were mounted onto gelatin-coated slides using Vectashield mounting medium with DAPI (product code #H-1200, Vectashield, Burlingame, CA, USA).

A LEICA TCS SP5 confocal laser scanning microscope (Leica Microsystems CMS GmbH, Mannheim, Germany) equipped with 20X or 63X objective (z step of 1.2 µm or 0.5 µm) was used to acquire confocal scans keeping all parameters constant. Image analyses were conducted on z-stacks projections on the CA1 area, the region of interest, using Image J (National Institute of Health, http://rsb.info.nih.gov/ij) (accessed on 30 November 2021).

Quantitative analyses on neurons and astrocytes were brought about in CA1 stratum pyramidalis (SP) or stratum radiatum (SR) on confocal microscopy z-projection of 10 consecutive z scans (20X objective, z step 1, 2 µm, total thickness 12 µm). Quantification of CA1 thickness, and of high-density nucleus (HDN) neurons, large HDN neurons, and low-density nucleus (LDN) neurons was performed in accordance with Landucci et al. [25]. GFAP immunofluorescence in CA1 SP or SR was detected from the number of positive pixels above a threshold level in each confocal microscopy z-projections using Image J with the threshold tool [26]. Astrocytes branches length was measured in CA1 SP or SR in accordance with Cerbai et al. [27].

### 2.6. Statistical Analysis

Data are presented as means ± SEM of n experiments. The statistical significance of differences between PI fluorescence intensities and Western blot was analysed using one-way ANOVA with a post hoc Dunnett w-test for multiple comparisons. Immunohistochemistry data were statistically analysed by one-way ANOVA followed by the Newman–Keuls multiple comparison test. All statistical calculations were performed using GRAPH-PAD PRISM v. 8 for Windows (GraphPad Software, San Diego, CA, USA). A probability value (*p*) of < 0.05 was considered significant.

## 3. Results

### 3.1. Acute and Prolonged Administration of Δ9-Tetrahydrocannabinol and Cannabidiol Do Not Produce Death in Mature Organotypic Hippocampal Slices

After 10–14 days in culture, the organotypic hippocampal slices reached maturation and were used for anatomical, molecular, and electrophysiological studies [28]. In this study, we analysed the effects of cannabinoids; in particular, the slices were treated with THC and CBD and the Cornu Ammonis areas CA1 region was evaluated for damage using PI fluorescence (Figure 1B). Quantitative analysis of hippocampal slices exposed for 24 h (Figure 1C) or 72 h (Figure 1D) to 1 µM THC or 10 µM CBD showed that these drugs did not induce injury in the CA1 region in this model compared with the exposure to maximal injury represented by 10 mM glutamate (24 h), which was used as a positive control (Figure 1B bottom left) [25,29].

### 3.2. Effect of THC and CBD on Synaptophysin and PSD95 Levels

Since in our slices we had previously observed functional alterations even in the absence of morphological damage [28], we examined the expression of a pre-synaptic and a post-synaptic protein following prolonged incubation with either cannabinoid.

**Figure 2:**
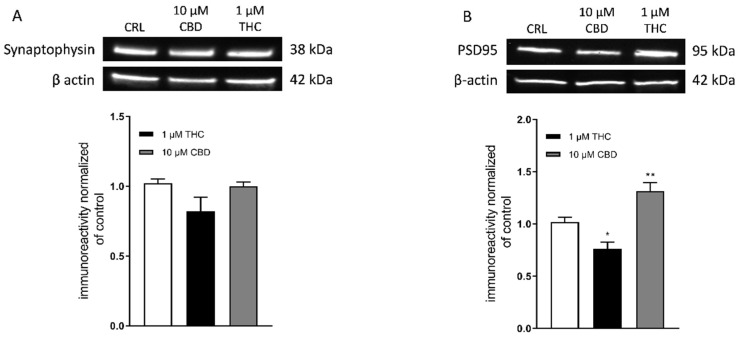
Effects of prolonged incubation with THC or CBD on synaptophysin and PSD95 expression in organotypic hippocampal slices. (**A**,**B**) Illustrative blots using antibodies directed against synaptophysin (Top (**A**)) and PSD95 (Top (**B**)) and β-actin ((**A**) and (**B**) centre). Quantitative analysis of WB bands shows no significant changes in synaptophysin levels in all experimental conditions (Bottom (**A**)). Incubation with 1 µM THC for 72 h caused a significant decrease in PSD95 levels, while 10 µM CBD for 72 h induced significant increase in PSD95 levels (Bottom (**B**)). Bars represent the mean ± SEM of at least four experiments. * *p* < 0.05, ** *p* < 0.01 vs. CRL (ANOVA + Dunnett’s w-Test).

We performed Western blotting analysis in homogenates of organotypic hippocampal slices exposed to THC (1 µM) or CBD (10 µM) for 72 h using antibodies directed against specific pre- and post-synaptic proteins (Figure 2). We found that THC or CBD did not significantly alter the levels of synaptophisin (Figure 2A) compared with the control slices. On the contrary, THC significantly decreased, while CBD increased PSD95 protein levels in the hippocampal homogenates (Figure 2B). These data are an indication that cannabinoids modify post-synaptic but not pre-synaptic proteins.

### 3.3. Prolonged THC or CBD Exposure Effects on Neuronal Viability

In order to ascertain whether the synaptic protein alterations translate into morphological modifications, we assessed the effects of either cannabinoid administration on pyramidal neurons in CA1 hippocampus of organotypic slices treated for 24 h or 72 h with 1 µM THC or 10 µM CBD. Neurons were immunostained with anti-NeuN antibodies and were visualised with confocal microscopy.

**Figure 3:**
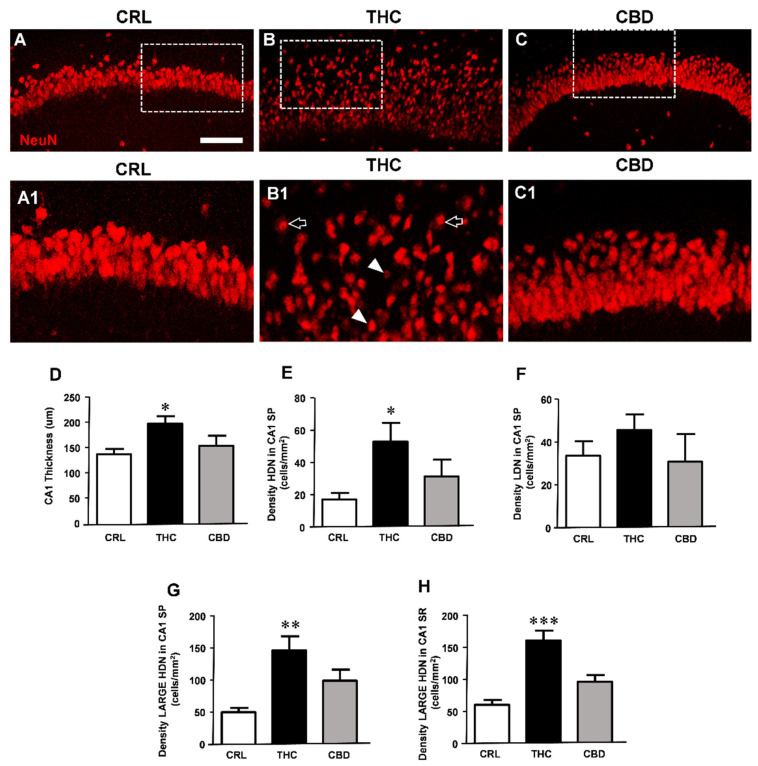
Prolonged THC or CBD exposure effects on neuronal viability. (**A**–**C**) Confocal microscopy images of NeuN immunostaining of neurons in CA1 SP and SR of a CRL (**A**), THC (**B**), and CBD slice (**C**) acquired with a 20X objective. A–C scale bar: 75 µm. (**A1**–**C1**) Enlargements of dotted areas of the corresponding slice in (**A**–**C**). (**A1**): The image shows healthy neurons in CA1 SP. (**B1**): The image highlights the profound alteration of neurons caused by subchronic THC exposure. Arrowheads and open arrows point to HDN neurons and large HDN neurons, respectively. (**C1**): The image shows that CBD treatment did not alter neurons of CA1 SP, which show a healthy morphology. A1–C1 scale bar: 30 µm. (**D**–**H**) Quantitative analyses of morphological alterations in CA1. (**D**) Thickness of CA1. Statistical analysis: one-way ANOVA *p* < 0.01; * *p* < 0.05 vs. CRL, Newman–Keuls post hoc test. (**E**) Density of HDN neurons in SP. Statistical analysis: one-way ANOVA *p* < 0.05; * *p* < 0.05 vs. CRL, Newman–Keuls post hoc test. (**F**) Density of LDN neurons. Statistical analysis: one-way ANOVA, not significant. (**G**) Density of large HDN neurons in SP. Statistical analysis: one-way ANOVA *p* < 0.01; ** *p* < 0.01 vs. CRL, Newman–Keuls post hoc test. (**H**) Density of large HDN neurons in SR. Statistical analysis: one-way ANOVA *p* < 0.0001; *** *p* < 0.001 vs. CRL, Newman–Keuls post hoc test). Bars represent the mean ± SEM of 6–8 experiments.

No significant qualitative or quantitative alterations in neurons were observed in slices treated for 24 h with THC or CBD (data not shown). Nevertheless, the qualitative analysis of the immunostaining of NeuN-positive neurons (Figure 3A,(C1)) shows that treatment with THC for 72 h caused disorganisation of the CA1 stratum pyramidalis (SP) and complex morphological modifications in a large number of pyramidal neurons. As evidenced in panel (B1), enlargement of the dotted area in B, many neurons distributed within the thickness of the CA1 region had a pyknotic nucleus (HDN neurons, white arrowheads in (B1)). In addition, numerous neurons had enlarged and vacuolised cytoplasm and high-density nuclei (large HDN neurons, open arrows in (B1)) both in the SP and SR (not shown in the images). Less consistently, other neurons were karyorrhectic, lacking their nuclear staining (low-density nucleus (LDN), not shown in the images). Thus, immunostaining for NeuN demonstrated that CA1 pyramidal neurons incubated for 72 h with THC showed signs of morphological alterations, compared with neurons in control slices. The effect of CBD on neuronal viability at the concentration and time of exposure used was less consistent in comparison to that of THC.

All the qualitative data were confirmed by quantitative analyses performed measuring the thickness of CA1 SP and the density of HDN, large HDN, and LDN neurons in SP and SR of control slices and of slices exposed for 72 h to THC or CBD (Figure 3D). We found that treatment with THC caused thorough disorganisation of CA1 SP. Pyramidal neurons were less densely packed throughout the thickness of CA1 SP, which increased significantly by about 44% in THC-treated slices (one-way ANOVA; * *p* < 0.05 vs. controls, Newman–Keuls post hoc test). CBD exposure did not increase significantly the CA1 SP layout (thickness +12%, n.s.).

Furthermore, THC exposure caused neuronal alterations, as shown by appearance of pyknotic (HDN) neurons in CA1 SP and of large HDN neurons in both SP and SR. Quantitative analysis demonstrated that treatment with THC for 72 h at the dose of 1 µM significantly increased the proportion of HDN neurons in SP (+212% vs. controls, * *p* < 0.05 THC vs. CRL, one-way ANOVA and Neuman–Keuls post hoc test, Figure 3E) and large HDN neurons in SP (+195% vs. controls, ** *p* < 0.01 THC vs. CRL, Figure 3G) and in SR (+167% vs. controls, *** *p* < 0.001 THC vs. CRL, Figure 3H). The effect of THC on the occurrence of LDN neurons was not significant (+36%, n.s., Figure 3F). Furthermore, in CA1 SP of THC-treated slices, the percentage of HDN/total neurons increased by 211%, and the percentage of large HDN/total neurons increased by 148% in SP and by 127% in SR, in comparison to control slices (all * *p* < 0.05, THC vs. CRL, not shown in the Figure). CBD treatment had no significant effect on neuronal morphological modifications in CA1 SP and SR. Indeed, the density of HDN, large HDN, and LDN neurons was not significantly different from controls in both SP and SR (Figure 3E–H). In conclusion, 72 h administration of THC, but not of CBD, caused significant tissue disorganisation and morphological alterations in CA1 pyramidal neurons.

### 3.4. Effects of THC or CBD Exposure on Astrocytes Viability

**Figure 4:**
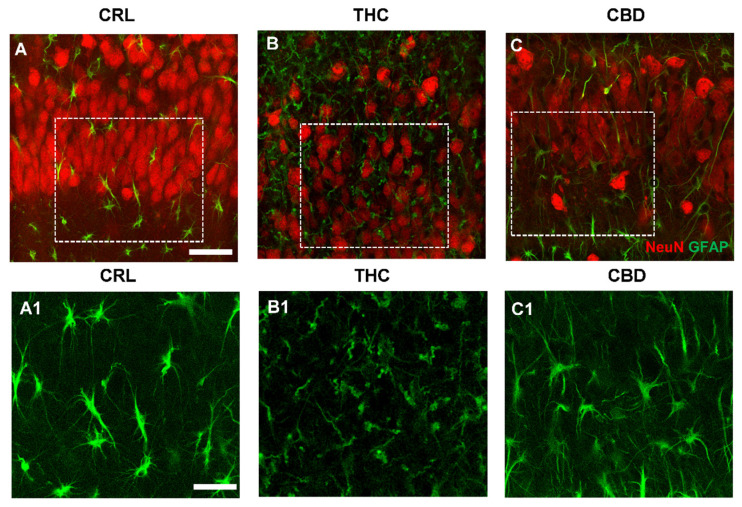

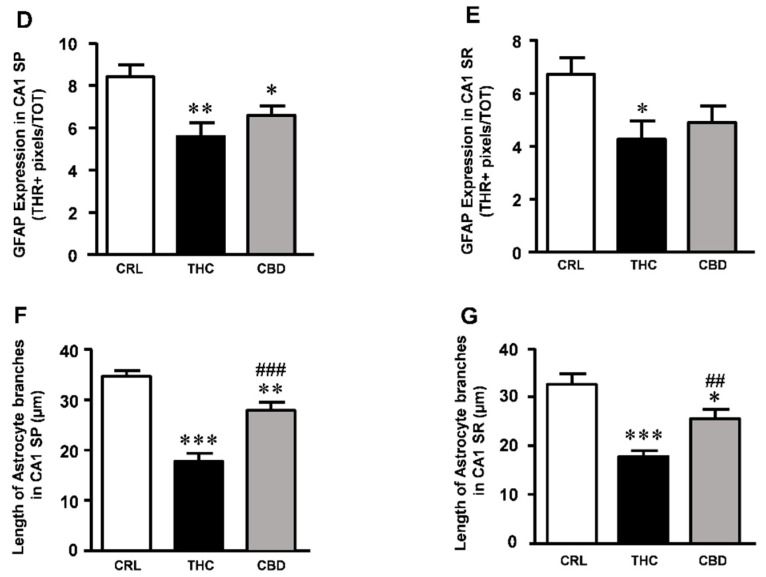
Effects of THC or CBD exposure on astrocytes viability. (**A**–**C**) Confocal microscopy images of immunostaining of astrocytes (GFAP antibody, green) and neurons (NeuN antibody, red) in CA1 SP of a CRL (**A**), THC (**B**), and CBD slice (**C**) acquired with a 63X objective. Scale bar: 20 µm. (**A1**–**C1**) Enlargements of the corresponding dotted areas in (**A**–**C**) showing astrocytes in a CRL (**A1**), THC (**B1**), and CBD slice (**C1**). Scale bar: 15 µm. (**A1**): The enlargement shows astrocytes with a healthy morphology in CA1 SP. (**B1**): The enlargement shows the profound morphological alterations of astrocytes caused by the subchronic THC exposure. (**C1**): The image shows that after CBD treatment, morphological alterations of astrocytes are less evident. (**D**) Quantitative analysis of GFAP expression in CA1 SP. Statistical analysis: one-way ANOVA *p* < 0.01; * *p* < 0.05 vs. CRL; ** *p* < 0.01 vs. CRL, Newman–Keuls post hoc test. (**E**) Quantitative analysis of GFAP expression in CA1 SR. Statistical analysis: one-way ANOVA *p* < 0.05; * *p* < 0.05 vs. CRL, Newman–Keuls post hoc test. (**F**) Quantitative analysis of astrocytes branches length in CA1 SP. Statistical analysis: one-way ANOVA *p* < 0.0001; *** *p* < 0.001 vs. CRL; ** *p* <0.01 vs. CRL; ^###^ *p* < 0.001 vs. THC, Newman–Keuls post hoc test. (**G**) Quantitative analysis of astrocytes branches length in CA1 SR. Statistical analysis: one-way ANOVA *p* < 0.0001; *** *p* < 0.05 vs. CRL; * *p* < 0.05 vs. CRL, ^##^ *p* < 0.01 vs. THC, Newman–Keuls post hoc test. Bars represent the mean ± SEM of 6–8 experiments.

No significant qualitative or quantitative alterations in astrocytes were observed in slices treated for 24 h with either cannabinoid (data not shown). The effect of 72 h exposure to THC or CBD was also evaluated on astrocytes viability in CA1 SP and SR of organotypic slices. Figure 4A–C show the merged confocal images of astrocytes (green) and neurons (red), and panels (A1–C1) show the enlargements of astrocytes in the corresponding dotted areas in A–C. Qualitative analyses indicate that 72 h THC exposure modified astrocytes morphology in CA1. Indeed, after prolonged treatment with THC, the branches of astrocytes in CA1 SP, compared with those of astrocytes in control and CBD-treated slices, appeared twisted and swirling, highly fragmented, and had lost their most distal processes (Figure 4(B1)), a modification named clasmatodendrosis by Cajal [30]. Furthermore, in THC-treated slices, the immunostaining of GFAP in astrocytes was less intense, as shown by the quantitative data reported in the graph of Figure 4D,E. Quantitative analyses demonstrated that 72 h exposure to THC significantly decreased GFAP expression both in CA1 SP (−34% vs. controls, ** *p* < 0.01 THC vs. CRL, one-way ANOVA and Neuman–Keuls post hoc test) and SR (−36% vs. controls, * *p* < 0.05 THC vs. CRL). The effect of CBD was statistically significant only in SP (−29% vs. controls, * *p* < 0.05 CBD vs. CRL), but not in SR (n.s.).

Quantitative analysis demonstrated that in THC-treated slices, the principal branches of astrocytes were significantly shorter in the SP (−48% vs. control; *** *p* < 0.001 THC vs. CRL, one-way ANOVA and Newman–Keuls multiple comparison test, Figure 4F) and in the SR (−45% vs. control; *** *p* < 0.001 THC vs. CRL, Figure 4G) compared with control slices. In slices treated for 72 h with CBD, the effect was less evident. Indeed, in the SP of CBD-treated slices, principal branches of astrocytes were 19% shorter than in control slices (** *p* < 0.01 CBD vs. CRL, and ^###^ *p* < 0.001 CBD vs. THC, Figure 4F), and in the SR they were 21% shorter than in control slices (* *p* < 0.05 CBD vs. CRL, and ^##^ *p*< 0.01 CBD vs. THC).

Lastly, panel (B1) clearly shows that prolonged THC exposure modified spatial tissue distribution of astrocytes, possibly mirroring the tissue disorganisation described above.

## 4. Discussion

The main finding of this study is that a prolonged incubation (72 h) with THC and CBD did not produce significant cell death in organotypic hippocampal slices but induced alteration in the expression of synaptic proteins and morphological alterations in neurons and astrocytes. In particular, the expression of PSD95 was reduced following incubation for 72 h with THC, while it increased following prolonged incubation with CBD. In corroboration of this finding, THC but not CBD caused disorganisation of the CA1 stratum pyramidalis (SP) and complex morphological modifications in a large number of pyramidal neurons and in astrocytes.

To explore the mechanisms underlying THC-induced neurobehavioral alteration observed in many previous studies, we exposed rat organotypic hippocampal slices to 1 µM THC or 10 µM CBD for 24 h or 72 h. At the end of these two time points, the slices were incubated with PI to detect cell death [23]. Our data of cellular death are in accordance with a study by Kreutz and colleagues [31], in which they exposed hippocampal slices cultured to 0.03 to 15 µM THC for 3 days and observed that THC-treated unlesioned slices contained almost no PI-positive degenerating neurons. In this study, we observed that exposure for 72 h with THC or CBD did not induce neuronal death observed by PI. Nevertheless, we found that 72 h administration of THC, but not of CBD, significantly increased neuronal morphological alterations, as demonstrated by the significantly higher proportion of pyknotic (HDN and large HDN) neurons. When we analysed the effects of cannabinoids incubation on the expression levels of pre- and post-synaptic proteins in hippocampal slices, we observed that exposure to 24 h of treatment did not induce alteration, whereas 72 h of incubation with THC caused a significant reduction in the expression of PSD95 while CBD evoked a significant increase. These data are in accordance with those observed by Rubino and colleagues [19], who showed that in rats treated for 10 days with THC, the post-synaptic marker PSD95 decreases and spatial memory is impaired. Chronic exposure to CP55,940, a synthetic cannabinoid agonist, alters both the morphology of pyramidal neurons and expression of PSD95 protein in the prefrontal cortex (PFC) and induces plasticity changes in the hippocampus–PFC network of adult rats [9]. Increased expression of synaptophysin and PSD95 in the medial prefrontal cortex (mPFC) and elevated BDNF levels in both mPFC and hippocampus after a single injection of cannabidiol are observed in Swiss mice and in Flinders Sensitive and Flinders Resistant Line (FSL/FRL) rats, which translates into acute antidepressant effects [32]. The increase in PSD95 is proposed as one of the possible mechanisms of action for CBD anti-stress effects [33]. When zebrafish embryos are exposed to ∆9-THC or CBD for 5 hours during gastrulation, embryos exhibit reduced heart rate, axial malformations, and shorter trunks [34]. Cannabinoid treatment alters synaptic activity at neuromuscular junctions (NMJs) [34].

Neurodegenerative diseases are usually studied from a neuronocentric point of view, but it is becoming evident that modifications of astrocytes and other glia cells are involved in neurodegeneration and CNS disorders. The importance of the role of astrocytes in neurodegeneration, whether damaging or protective, is still unknown. Nevertheless, it has been postulated that diverse mechanisms, as a change in the release or uptake of gliotransmitters such as glutamate, clasmatodendrosis, and astrocytes death, indirectly contribute to neuronal loss [35,36,37,38,39]. Therefore, a part of our study was aimed at understanding whether morphology and viability of astrocytes are modified by 72 h exposure to THC or CBD early after the end of drug exposure and may be reflected in neuronal modifications. It has been shown in the hippocampus that neuronal-released endocannabinoids activate astrocytic CB1 receptors, leading to an increase in intracellular Ca^2+^ release from internal stores [40,41] and, consequently, the release of gliotransmitters, such as glutamate, ATP and D-serine [42,43,44]. It has also been demonstrated in spinal cord astrocytes stimulated by cannabinoids through CB1 receptors activation that intracellular Ca^2+^ and release of 2-AG increase [45]. In our experiments, we found that both THC and CBD exposure for 72 h caused significant modifications of astrocytes morphology, possibly reflecting functional alterations, as evidenced by the decrease in GFAP expression and morphological alterations of astrocytes branches. In both CA1 SP and SR of THC-treated slices, astrocytes showed marked signs of clasmatodendrosis, an irreversible astrocytic degeneration characterised by the dissolution of their branches [30]. Clasmatodendrosis has been linked to autophagy [46,47], suggesting that it may represent an additional mechanism of astrocytic death. Furthermore, branches of clasmatodendrotic astrocytes are shorter and lose their more distal processes and endfeet, causing less coverage of brain vessels. Therefore, clasmatodendrotic astrocytes may lose their trophic and supportive functions to neurons. In addition, in the healthy brain, astrocytes regulate the formation, maturation, and plasticity of synapses [48,49,50,51], controlling the development and maintenance of neural circuits [52,53,54]. Astrocytes, mediating the functionality of synapses [55], are involved indirectly in memory mechanisms [48,49,56].

It is known that astrocytes control the formation, maturation, and plasticity of synapses by secreting many proteins that regulate synaptic formation, such as thrombospondins, hevin, and solid-phase attachment of red cells (SPARC) [50,51]. In addition, as pointed out above, healthy astrocytes envelope synapses with their processes and are indispensable for neurotransmitter homeostasis, the release of gliotransmitters, and the maintenance and maturation of synapses [57,58]. In addition, astrocytes control the levels of GABA and glutamate at the synapses, thus mediating the functions of the so-called tripartite synapse [55]. It is therefore plausible that astrocytes clasmatodendrosis caused by continuous exposure to THC, which leads to spatial disorientation of astrocytes, and disruption of astrocytic syncytium, may decrease maintenance of healthy synapses and synaptic connectivity and can play a role in decreasing neuronal homeostasis [49,59], partly explaining the data obtained in our experiments. The involvement of astrocytes in THC-dependent memory deficits has been recently demonstrated [60]. THC exposure in genetically predisposed adolescences seems to cause synergistical activation of NF-kB–COX-2 signalling in astrocytes, increased secretion of glutamate, decreased parvalbumin-positive pre-synaptic boutons around pyramidal neurons of the CA3 area of the hippocampus, and memory deficits.

Nevertheless, in CBD-treated slices astrocytes morphology was not significantly different to that of control astrocytes, and the length of their branches was intermediate between control and THC-treated slices. No modifications typical of clasmatodendrosis were evident. These data demonstrated that 72 h treatment of slices with THC was a sufficient time to evoke strong degeneration of astrocytes, while incubation with CBD for the same time had a milder effect. CBD has been shown to play a therapeutic role in inhibiting reactive astrogliosis in various in vivo models of brain disorders. For instance, CBD reduces GFAP overexpression in astrocytes in the pilocarpine-induced epileptic model through the activation of the PI3K signalling pathway [61]. CBD also suppresses the expression of astrogliosis-marker proteins via PPAR activation and NF-kB inhibition in an amyloid mouse model of AD [62]. Nevertheless, it will be of interest to study the effect of CBD administration at longer time points.

A recent hypothesis postulates that in physiopathological conditions, astrocytes exist as a continuum of heterogeneous, mixed populations [63,64,65,66,67], depending not only on the type of insult but also on the brain region and the time after the insult. The alterations in astrocytes morphofunctional states may cause modifications of brain homeostasis by impairment of extracellular glutamate buffering, reducing the supply of nutrients to neurons, thus contributing to the increased damage to neurons [68], as occurred in our model after subchronic THC exposure. Longer exposures to cannabinoids may cause further damage to astrocytes, suppressing their functionality and increasing neuronal vulnerability. All these effects at length may possibly be at the basis of neuronal degeneration [38,69].

The pharmacological targets of THC and CBD are known to be diverse [70,71,72]. In particular, THC is known to interact as an agonist on CB1 and CB2 receptors, whereas CBD interacts with multiple targets, including an agonist-like effect on the peroxisome proliferator-activated receptor γ (PPARγ), transient potential receptors V (TRPV1, TRPV2), and indirectly on CB1 and CB2 receptors, by inhibiting the enzyme fatty acid amide hydrolase that degrades anandamide, with an increase in anandamide concentration. In addition, CBD exerts an effect as an orthosteric agonist on 5-HT1A receptor. Hence, these different mechanisms of action of THC and CBD may explain the differences in their effects that we have observed in this study. As for the effects in organotypic hippocampal slices, we have recently shown that in our model, THC appears to act mainly on CB1 receptors, whereas the effects of CBD were blocked by TRPV2, 5-HT1A, and PPARγ receptor antagonists, but not by antagonists of CB1 receptors [25].

## 5. Conclusions

In conclusion, our results show that prolonged exposure to THC or CBD induced different and sometimes opposite effects in the hippocampus. In particular, THC reduced while CBD increased PSD95 levels. Furthermore, THC but not CBD caused disorganisation of the hippocampus layout and brought about complex morphological modifications in a large number of pyramidal neurons and astrocytes. Therefore, the appropriate medicinal use of THC with chronic treatment should be considered with more attention, particularly in adolescents.

## Data Availability

Our own data presented in this study are available on request from the corresponding author.
